# Environmental extreme temperature and daily preterm birth in Sabzevar, Iran: a time-series analysis

**DOI:** 10.1186/s12199-018-0760-x

**Published:** 2019-01-05

**Authors:** Danial Mohammadi, Elham Naghshineh, Alireza Sarsangi, Mohammad Javad Zare Sakhvidi

**Affiliations:** 1 Occupational Health Research Center, School of Public Health, Shahid Sadoughi University of Medical Sciences, Yazd, Iran; 2 Department of Occupational Health, School of Public Health, Shahid Sadoughi University of Medical Sciences, Yazd, Iran; 3 Department of Obstetrics and Gynecology, School of Medicine, Isfahan University of Medical Sciences, Isfahan, Iran; 4 Department of Remote Sensing and GIS, Faculty of Geography, University of Tehran, Tehran, Iran

**Keywords:** Environmental exposure, Heat stress, Premature birth, Temperature

## Abstract

**Objectives:**

Most of the studies on the effect of heat stress on preterm birth (PTB) are conducted in temperate climates. Evidence on this effect in hot and arid countries with low and middle income is limited. This paper describes the short-term effect of exposure to the hot and cold environment on a daily number of PTB in Iran.

**Methods:**

The daily number of PTB was obtained from all hospitals of the city. Meteorological and air pollution data from 2011 to 2017 were obtained from a metrological station in the city. A semi-parametric generalized additive model following a quasi-Poisson distribution with the distributed lag non-linear model was selected as a modeling framework for time-series analysis to simultaneously model the short-term and lagged effect of heat stress on PTB in the Sabzevar city.

**Results:**

The minimum and maximum daily temperature were − 11.2 and 45.4 °C respectively. The highest risk estimate at extreme cold temperature was found for apparent temperature (relative risk (RR) 1.83; 95% CI 1.61: 2.09). This pattern was seen for both models. For extreme hot temperatures, the model with mean temperature showed the highest risk increase for both the main model and air pollution adjusted model (RR 1.36; 95% CI 1.25: 1.49). The lowest risk estimate in extremely cold conditions was found in the model with mean temperature. However, for extremely hot temperature conditions, the lowest risk estimate was found for both maximum and apparent temperature.

**Conclusion:**

Obstetricians working in semi-arid areas should be aware of the influence of environmental extreme temperature on the incidence of PTB.

**Electronic supplementary material:**

The online version of this article (10.1186/s12199-018-0760-x) contains supplementary material, which is available to authorized users.

## Introduction

About 5 to 18% of all births are preterm. At 2010, approximately 11.1% of all live births (149 million births) were preterm birth (PTB). PTB is the second most important direct cause of child mortality after pneumonia in the world [1]. PTB trend increased from 1990 to 2010 in most countries. In addition to its effect on child mortality and morbidity, PTB imposes a huge economic burden on the families and societies later in life because of its lifelong adverse effects [2, 3]. Several well-known factors (mainly maternal and behavioral characteristics) such as maternal tobacco use, age, body mass index, hypertension, and infection are recognized as the classic risk factors of PTB [4–6]. However, recently, the role of environmental and occupational factors in the increasing risk of PTB has received more attention [7–9]. Air pollution and meteorological conditions are among the recently highlighted risk factors of PTB [10, 11].

The global warming and climate change intensified the importance of this problem. Heat stress generally is defined as an imbalance between heat production and heat loss of the human body [12]. The cyclic pattern of a daily number of PTB with the meteorological parameters of preceding and index day of delivery was reported in several previous studies [4, 11, 13]. Recent studies proposed the short-term effect of exposure to environmental thermal stress on PTB [13]. Several other studies found no significant association between temperature and preterm birth [14–16]. The exact physiological mechanism of effect of temperature on PTB is not clear. Generally, pregnant women have lower thermoregulatory capacity in comparison with men. In pregnant women, the decreases in the ratio of body surface area to body mass index lead to less heat loss capacity. On the other side, fetal growth increases the internal heat production. This situation leads to susceptibility of pregnant women to heat stress [17].

Most studies on the effect of temperature on PTB conducted in locations with moderate temperature. It seems that parts of observed controversy in the available studies are because of the geographic location of the study area. In addition, the evidence of temperature effects on PTB in more arid countries and also countries with low and middle income is limited. In this study, we used a 7-year data on preterm birth and also daily values of different meteorological parameters to examine the hypothesis of an association between environmental heat stress and PTB. We also used apparent temperature (AT) in addition to mean and maximum daily temperature in the models to compare the sensitivity of predictions according to different meteorological parameters.

## Method

### Study area and population

**Fig. 1:**
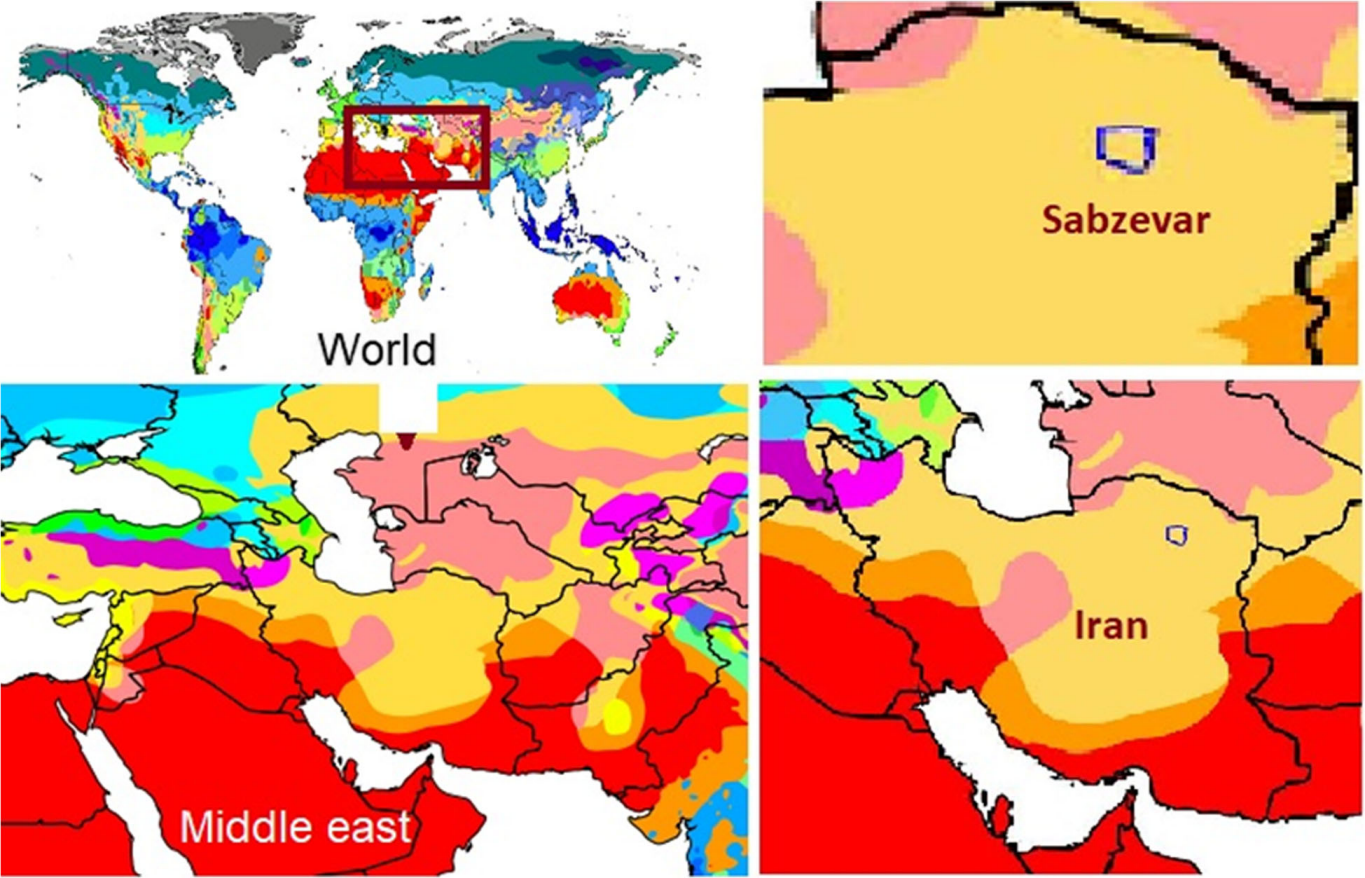
Location of the study area (Sabzevar city) in I.R. Iran according to Köppen climate classification scheme symbols

Sabzevar city is located at northeastern Iran with a resident population of 231,557 according to the 2011 census. It is located in a hot and dry region (coordinates: 36° 12′ N 57° 35′, elevation: 977.6 m) with arid climate and four distinct seasons according to the Köppen climate classification (Fig. 1) [18]. The study was approved by the Ethics Committee of the Shahid Sadoughi University of Medical Sciences (Ethics Committee approval number: IR.SSU.SPH.REC.1397.017). The PTB data in the Sabzevar city was collected according to the health information system (HIS) data from all four hospitals of the city from 21 March 2011 to 30 June 2017. All records which were coded “O60” according to the International Classification of the Diseases 10th version (ICD-10) were considered as a PTB. The date of hospital admission because of contraction was used as a date of analysis in this study. The PTB was defined as those births below complete 37 weeks of gestation. Gestational age was determined according to the first-time NP ultrasonography and last menstrual period (LMP) data. However, in the case of difference between these two indexes, the LMP was used as a criterion for determination of gestational age. When the difference between NP ultrasonography and the LMP was more than 1 week, the result of NP was considered for calculation of gestational age.

Raw meteorological data including mean and maximum temperature (°C), relative humidity (%), wind velocity (km/h), and precipitation (mm/day) in daily basis were collected from the Sabzevar climatology department. Data on daily air pollution status were obtained in a form of a 5-point ordinal scale based on daily meteorological visibility (0: normal condition; 1: small amount of dust pollution; 2: medium pollution; 3: visible distance less than 1000; 4: visible distance less than 200). In addition to the mean and maximum daily temperature, apparent temperature (AT) index was also calculated based on the available data and used in the modeling frameworks. Heat stress indexes combine different meteorological parameters to produce a single metric to represent the thermal burden imposed on a human body at different situations [12]. The AT index was calculated according to the following equation:

$$ \mathrm{AT}=T+0.33\ \mathrm{VP}-0.7\ V-4 $$

where *T* is dry bulb temperature (°C), VP is vapor pressure (hPa), and *V* is air velocity (m/s).

### Statistical analysis

Previous studies have shown a non-linear and lagged effect of environmental temperature on the PTB. Therefore, knowing over-dispersions of data, in this study, a semi-parametric generalized additive model (GAM) following a quasi-Poisson distribution with distributed lag non-linear model (dlnm) was selected as a modeling framework for time-series analysis. In this framework, it is possible to simultaneously model the short-term and lagged effect of temperature on the daily number of PTB in the city. The dlnm is a modeling framework which allows to simultaneously investigate non-linear and delayed association between the predictors and an outcome. A dependency is defined as exposure-lag-response association in a bidimensional (crossbasis matrix) framework [19]. The dlnm was originally proposed to study the health effects of temperature. The immediate effect of predictor(s) on the outcome can be obtained at lag 0. Different maximum significant lag periods were reported across studies. In this study, we selected a maximum 14-day lag response as a maximum lag time for the outcome. A model was constructed to assess the effect of heat stress on PTB, while adjusting for the effect of the day of the week, raining, holidays, air pollution, and long time and seasonal trend. Therefore, a general form of a time-series model applied in this study to model outcome (*E*(*Yt*)) was as below:

$$ \log \left[E(Yt)\right]=\upalpha +\upbeta \mathrm{cbTemperature}\ \left(\mathrm{fun}=\mathrm{ns},\mathrm{df}=5;=\mathrm{lag}:7,\mathrm{fun}=\mathrm{ns},\mathrm{df}=4\right)+\updelta \mathrm{cbDust}\left(\mathrm{fun}=\mathrm{integer};=\mathrm{lag}:7,\mathrm{fun}=\mathrm{ns},\mathrm{df}=4\right)+\upmu \mathrm{DOW}+\upgamma \mathrm{dayofyear}+\upsigma \mathrm{Rain}+\upvartheta \mathrm{Holiday} $$

In this model, *α* is the intercept and *ß*, *δ*, *γ*, *σ*, *ϑ*, and *μ* are coefficients. “cb” is a crossbasis object of heat stress and lag time. We included the relative humidity in the models for maximum and mean temperatures. However, we did not consider the relative humidity in the models with AT as a predictor, because of the inclusion of relative humidity (as vapor pressure) directly in the calculation of AT. Inclusion of the relative humidity in the models with AT will lead to collinearity. Different *df*s reported for association between environmental temperature and health outcomes in previous studies. In this study, we used a range of *df*s reported across previous studies. After initial analysis with different *df*s, those with lowest cross-validation score (CVS) were used to select the final model [20–22]. For heat stress or temperature space, the natural cubic spline with 4–5 degrees of freedom (*df*) was reported. For lag time, also natural cubic spline with *df* 5 was selected. For time space, the natural cubic spline with 4 to 7 *df*s per year was reported. Day of week (DOW) and air pollution (Dust) entered into the study as a categorical variable. Crossbasis matrix for relative humidity was also constructed using natural cubic spline with *df* 3. Holidays were entered as a binary variable in the model to adjust the public holidays according to the Iranian calendar.

We added additional analysis to our study defining a new variable computed from subtracting maximum temperature from minimum temperature for each day (defined as daily temperature variation). We used 0 °C as a centering point in these models (because it means no change in the daily temperature). In a separate analysis, we used a heatwave definition (mean daily temperature > 90th percentile for ≥ 2 consecutive days) and built a model based on occurrence of heatwave as a predictor variable in the models.

All data analyses were conducted using R software (version 3.3.0). The “dlnm” package was used to fit dlnm [19].

## Results

During the study period from 2011 till 2017, a total of 3140 cases of PTB (mean daily reported case: 2.16 ± 1.34) were recorded in the city. Descriptive statistics on the daily number of PTB, and meteorological factors according to year, month, the day of the week, holidays, and working days are reported in Table 1. The highest and the lowest daily frequency of PTB was observed in the year 2017 (3.07 ± 1.55) and 2012 (1.95 ± 1.19) respectively. However, there was no significant difference between the frequencies of PTB according to the study years. Saturday and Friday respectively had the highest and the lowest number of PTB.

**Table 1:** Descriptive statistics on the daily number of PTBs, temperature, relative humidity, wind, daily rain and dust code (2011–2017)

Variable	All PTBs		Temperature			Relative humidity			Average wind (mean ± SD)	Average rain (mean ± SD)	Average dust code (mean ± SD)
	Total number	Daily average ± SD	Average maximum temperature (mean ± SD)	Average minimum temperature (mean ± SD)	Average temperature (mean ± SD)	Average maximum RH (mean ± SD)	Average minimum RH (mean ± SD)	Average (mean ± SD)			
Year											
2011	415	2.23 ± 1.81	28.24 ± 10.73	15.06 ± 9.01	28.24 ± 10.73	50.11 ± 24.58	19.35 ± 17.55	34.73 ± 20.14	8.13 ± 2.42	0.68 ± 2.84	0.68 ± 0.93
2012	513	1.95 ± 1.19	24.08 ± 11.29	11.44 ± 9.49	24.09 ± 11.29	55.00 ± 25.31	23.92 ± 17.53	39.46 ± 20.73	7.54 ± 2.51	0.54 ± 2.47	0.85 ± 0.92
2013	488	2.08 ± 1.18	25.77 ± 10.64	12.44 ± 9.42	25.77 ± 10.64	52.82 ± 23.10	20.20 ± 14.04	36.51 ± 17.72	8.03 ± 2.58	0.27 ± 1.75	0.69 ± 0.94
2014	448	2.03 ± 1.21	25.01 ± 11.45	12.04 ± 9.64	25.01 ± 11.45	53.16 ± 26.11	21.07 ± 16.15	37.12 ± 20.06	11.83 ± 3.79	0.44 ± 1.84	0.66 ± 0.97
2015	503	2.02 ± 1.03	25.59 ± 10.82	12.71 ± 9.10	25.59 ± 10.82	52.99 ± 23.83	22.34 ± 15.53	37.66 ± 19.05	11.85 ± 3.44	0.33 ± 1.42	1.13 ± 1.06
2016	518	2.21 ± 1.23	26.02 ± 10.26	12.68 ± 8.95	26.02 ± 10.26	53.53 ± 22.29	18.62 ± 12.15	36.08 ± 16.36	11.73 ± 3.52	0.37 ± 1.69	1.88 ± 1.03
2017	255	3.07 ± 1.55	22.73 ± 10.86	10.03 ± 9.15	22.73 ± 10.86	62.23 ± 21.93	22.82 ± 17.40	41.35 ± 19.53	11.72 ± 3.66	0.75 ± 2.81	2.56 ± 0.90
Month											
January	286	2.27 ± 1.42	11.42 ± 4.32	0.41 ± 3.37	5.92 ± 3.54	75.06 ± 15.28	34.35 ± 13.48	54.71 ± 13.24	7.78 ± 2.94	0.50 ± 1.72	1.15 ± 1.09
February	267	2.18 ± 1.28	13.11 ± 5.72	1.87 ± 4.57	7.49 ± 4.82	71.56 ± 16.46	33.71 ± 16.54	52.63 ± 15.63	9.02 ± 3.36	0.97 ± 3.18	1.30 ± 1.26
March	263	2.07 ± 1.04	18.64 ± 5.16	6.47 ± 4.41	12.55 ± 4.45	70.23 ± 16.58	28.40 ± 13.41	49.31 ± 13.16	10.68 ± 3.93	0.75 ± 2.62	0.93 ± 0.99
April	257	1.98 ± 1.13	26.19 ± 4.96	12.01 ± 3.89	19.09 ± 4.18	60.50 ± 19.49	19.11 ± 11.84	39.66 ± 14.65	11.27 ± 4.06	0.61 ± 2.15	0.87 ± 1.12
May	216	1.86 ± 1.13	32.69 ± 3.62	18.26 ± 3.10	25.47 ± 3.15	50.18 ± 19.07	13.76 ± 7.97	31.43 ± 12.83	11.65 ± 3.94	0.61 ± 2.52	1.29 ± 1.16
June	394	2.57 ± 1.24	37.24 ± 3.02	22.94 ± 2.85	30.08 ± 2.69	33.22 ± 14.06	9.35 ± 5.44	21.02 ± 9.10	11.94 ± 3.52	0.21 ± 1.75	1.67 ± 1.27
July	369	2.72 ± 1.41	38.67 ± 2.56	24.97 ± 2.40	31.82 ± 2.25	27.79 ± 10.20	9.12 ± 3.95	18.45 ± 6.56	11.54 ± 3.25	0.04 ± 0.51	1.81 ± 1.07
August	311	2.51 ± 1.95	37.03 ± 2.73	22.34 ± 2.60	29.69 ± 2.44	27.23 ± 10.35	9.16 ± 4.96	18.20 ± 7.22	10.77 ± 2.96	0.02 ± 0.29	1.35 ± 1.02
September	208	1.67 ± 0.73	33.80 ± 3.20	19.09 ± 2.65	26.45 ± 2.70	34.95 ± 13.46	10.69 ± 5.48	22.82 ± 8.92	9.79 ± 2.80	0.08 ± 1.02	0.83 ± 0.96
October	180	1.27 ± 0.51	26.19 ± 4.88	12.42 ± 3.84	19.30 ± 4.17	49.52 ± 17.84	18.32 ± 10.97	33.92 ± 13.56	9.47 ± 3.15	0.21 ± 1.14	0.48 ± 0.79
November	176	1.80 ± 1.00	16.18 ± 5.27	5.28 ± 3.83	10.73 ± 4.22	70.88 ± 18.32	33.88 ± 18.03	52.38 ± 17.13	8.38 ± 3.50	0.90 ± 3.05	0.62 ± 0.98
December	213	1.96 ± 1.37	11.48 ± 4.18	0.90 ± 3.54	6.19 ± 3.56	75.62 ± 16.66	36.75 ± 16.56	56.18 ± 15.57	7.59 ± 2.93	0.56 ± 2.47	0.99 ± 1.07
Day of week											
Sunday	437	2.08 ± 1.23	25.23 ± 11.19	12.35 ± 9.39	18.79 ± 10.18	53.92 ± 24.54	21.52 ± 16.16	37.63 ± 19.62	10.05 ± 3.64	0.45 ± 1.84	1.12 ± 1.18
Monday	434	2.16 ± 1.39	25.42 ± 11.14	12.31 ± 9.47	18.87 ± 10.21	53.22 ± 24.52	20.52 ± 15.66	36.79 ± 19.07	10.04 ± 3.90	0.43 ± 2.00	1.16 ± 1.31
Tuesday	449	2.09 ± 1.08	25.48 ± 11.08	12.31 ± 9.38	18.90 ± 10.12	52.99 ± 24.12	21.07 ± 16.19	36.93 ± 19.35	10.02 ± 3.64	0.39 ± 1.91	1.12 ± 1.10
Wednesday	485	2.29 ± 1.35	25.67 ± 10.95	12.55 ± 9.38	19.10 ± 10.06	54.48 ± 24.80	21.20 ± 16.06	37.72 ± 19.57	10.12 ± 3.92	0.79 ± 3.17	1.11 ± 1.12
Thursday	447	2.08 ± 1.07	25.54 ± 10.66	12.56 ± 9.24	19.06 ± 9.87	54.58 ± 24.63	21.08 ± 14.98	37.72 ± 18.94	10.07 ± 3.84	0.34 ± 1.75	1.09 ± 1.14
Friday	413	1.90 ± 0.90	25.52 ± 10.86	12.50 ± 9.23	19.01 ± 9.94	53.88 ± 23.68	21.30 ± 16.00	37.53 ± 19.01	10.15 ± 3.66	0.42 ± 2.07	0.99 ± 1.13
Saturday	475	2.47 ± 1.94	25.41 ± 10.86	12.53 ± 9.39	18.96 ± 10.00	53.05 ± 23.14	21.13 ± 15.39	37.03 ± 18.45	9.91 ± 3.36	0.36 ± 1.51	1.18 ± 1.16
Holiday											
Yes	631	1.96 ± 0.89	25.10 ± 10.76	12.14 ± 9.17	18.62 ± 9.85	54.96 ± 24.04	21.37 ± 15.95	38.11 ± 19.05	10.18 ± 3.72	0.48 ± 2.04	1.00 ± 1.11
No	2509	2.21 ± 1.42	25.57 ± 11.00	12.52 ± 9.39	19.04 ± 10.09	53.40 ± 24.22	21.05 ± 15.72	37.13 ± 19.15	10.02 ± 3.71	0.45 ± 2.11	1.14 ± 1.14

**Fig. 2:**
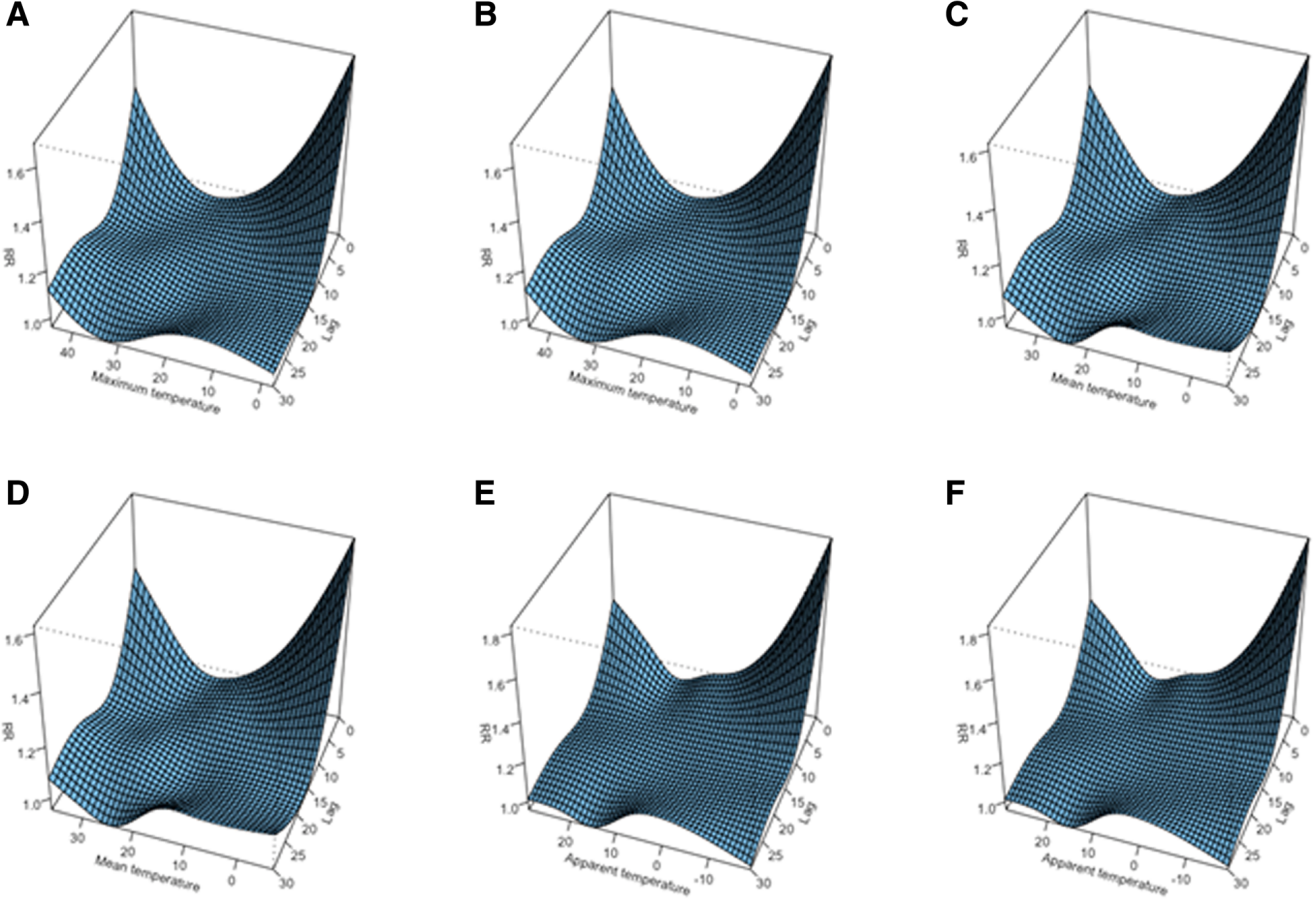
Risk estimates for preterm birth for different heat stress parameters at different lag times (**a**, **c**, and **e** are models without air pollution adjustment, whereas **b**, **d**, and **f** are for models with air pollution adjustment)

In the models with mean, maximum, and AT index which were adjusted for air pollution, we found a “U” shape non-linear relationship with PTBs at lags 0 to 3. At longer lags, the responses were flat (Fig. 2). A non-significant increase in relative risk (RR) was found for lags between 8 and 12 especially at elevated temperatures. The temperature effect was highest at lag 0, and the RR increased with increasing or decreasing the temperature at both extremes.

Tables 2, 3, and 4 show the RR estimates and 95% confidence intervals for a mean, maximum, and apparent temperature at 1st, 25th, 75th, and 99th percentiles in comparison with the median of each index over the different lag days. The risk estimates were significantly elevated at all selected percentiles. A decreasing trend in risk estimates at percentiles 1st, 25th, 75th, and 99th for all heat stress indexes was observed from index day of delivery to further lag days. However, the highest increase in risk was observed for extremely low temperatures. A similar pattern was found for a model without adjustment for air pollution. The highest risk estimate at extreme cold temperature was found for maximum, mean, and apparent temperature models. This pattern was also seen for air pollution-adjusted models. For extremely hot temperatures, the model with the mean temperature showed the highest risk increase for both main model and air pollution-adjusted model (RR 1.60; 95% CI 1.37: 1.86). The lowest risk estimate at extremely cold conditions was found in the model with apparent temperature. However, for extremely high-temperature conditions, the lowest risk estimate was found for both maximum temperatures.

**Table 2:** Relative risk (RR) estimates and 95% confidence intervals for preterm births and mean temperature. All RRs are relative to median value of mean temperature distribution (19.9 °C)

	Model 1				Model 2 (additionally adjusted for air pollution)			
	P1	P25	P75	P99	P1	P25	P75	P99
lag0	1.79 (1.58: 2.04)	1.13 (1.02: 1.25)	1.26 (1.13: 1.4)	1.66 (1.43: 1.92)	1.76 (1.54: 2)	1.13 (1.02: 1.25)	1.25 (1.12: 1.4)	1.6 (1.37: 1.87)
lag1	1.51 (1.4: 1.64)	1.09 (1.03: 1.17)	1.17 (1.09: 1.25)	1.44 (1.31: 1.58)	1.49 (1.37: 1.62)	1.1 (1.03: 1.17)	1.16 (1.08: 1.24)	1.39 (1.26: 1.54)
lag2	1.3 (1.23: 1.37)	1.06 (1.02: 1.11)	1.09 (1.04: 1.14)	1.26 (1.18: 1.34)	1.28 (1.21: 1.35)	1.07 (1.02: 1.11)	1.08 (1.03: 1.13)	1.22 (1.14: 1.31)
lag3	1.14 (1.07: 1.21)	1.04 (0.99: 1.08)	1.03 (0.98: 1.08)	1.12 (1.05: 1.2)	1.12 (1.06: 1.2)	1.04 (1: 1.09)	1.02 (0.97: 1.07)	1.09 (1.01: 1.18)
lag4	1.03 (0.96: 1.11)	1.02 (0.96: 1.07)	0.99 (0.93: 1.05)	1.03 (0.95: 1.12)	1.03 (0.95: 1.1)	1.02 (0.97: 1.08)	0.97 (0.92: 1.04)	1 (0.92: 1.1)
lag5	0.99 (0.92: 1.06)	1.01 (0.96: 1.07)	0.97 (0.91: 1.03)	0.98 (0.9: 1.07)	0.98 (0.91: 1.05)	1.01 (0.96: 1.07)	0.95 (0.9: 1.02)	0.96 (0.88: 1.05)
lag6	0.99 (0.94: 1.05)	1.01 (0.96: 1.06)	0.97 (0.92: 1.02)	0.97 (0.9: 1.04)	0.98 (0.92: 1.04)	1.01 (0.96: 1.06)	0.96 (0.91: 1.01)	0.96 (0.88: 1.03)
lag7	1.03 (0.98: 1.08)	1.01 (0.97: 1.06)	0.98 (0.94: 1.03)	0.98 (0.92: 1.04)	1.01 (0.96: 1.07)	1.01 (0.97: 1.05)	0.98 (0.93: 1.02)	0.98 (0.91: 1.05)
lag8	1.08 (1.01: 1.14)	1.02 (0.97: 1.07)	1.00 (0.95: 1.06)	1.01 (0.94: 1.09)	1.06 (0.99: 1.13)	1.01 (0.96: 1.06)	1.01 (0.96: 1.06)	1.01 (0.94: 1.09)
lag9	1.12 (1.04: 1.2)	1.03 (0.97: 1.09)	1.03 (0.97: 1.09)	1.05 (0.96: 1.14)	1.1 (1.02: 1.19)	1.01 (0.96: 1.07)	1.03 (0.97: 1.1)	1.05 (0.96: 1.15)
lag10	1.13 (1.05: 1.21)	1.03 (0.97: 1.08)	1.05 (0.99: 1.11)	1.07 (0.98: 1.17)	1.11 (1.03: 1.2)	1.01 (0.96: 1.07)	1.05 (0.99: 1.12)	1.08 (0.98: 1.18)
lag11	1.11 (1.04: 1.17)	1.02 (0.97: 1.07)	1.06 (1.01: 1.12)	1.09 (1.01: 1.17)	1.09 (1.03: 1.16)	1.01 (0.96: 1.05)	1.06 (1.01: 1.12)	1.09 (1.01: 1.17)
lag12	1.06 (1: 1.12)	1.01 (0.96: 1.05)	1.07 (1.02: 1.12)	1.09 (1.02: 1.17)	1.05 (0.99: 1.12)	1 (0.95: 1.04)	1.07 (1.02: 1.12)	1.09 (1.01: 1.17)
lag13	1 (0.91: 1.09)	0.99 (0.93: 1.06)	1.08 (1: 1.15)	1.09 (0.99: 1.2)	1 (0.91: 1.09)	0.99 (0.92: 1.05)	1.06 (0.99: 1.14)	1.08 (0.97: 1.2)
lag14	0.93 (0.81: 1.07)	0.98 (0.88: 1.08)	1.08 (0.97: 1.2)	1.09 (0.93: 1.27)	0.94 (0.81: 1.08)	0.97 (0.88: 1.08)	1.06 (0.95: 1.18)	1.07 (0.91: 1.26)

**Table 3:** Relative risk (RR) estimates and 95% confidence intervals for preterm births and Maximum temperature. All RRs are relative to median value of Maximum temperature distribution (26.9 °C)

	Model 1				Model 2 (additionally adjusted for air pollution)			
	P1	P25	P75	P99	P1	P25	P75	P99
lag0	1.78 (1.56: 2.03)	1.12 (1.01: 1.24)	1.18 (1.07: 1.3)	1.57 (1.38: 1.80)	1.75 (1.53: 1.99)	1.12 (1.01: 1.24)	1.18 (1.07: 1.3)	1.53 (1.33: 1.76)
lag1	1.54 (1.42: 1.67)	1.09 (1.03: 1.17)	1.11 (1.05: 1.19)	1.38 (1.26: 1.50)	1.52 (1.39: 1.65)	1.1 (1.03: 1.17)	1.11 (1.04: 1.18)	1.34 (1.22: 1.47)
lag2	1.35 (1.27: 1.42)	1.07 (1.02: 1.12)	1.06 (1.01: 1.1)	1.22 (1.15: 1.29)	1.33 (1.25: 1.41)	1.07 (1.03: 1.12)	1.05 (1.01: 1.1)	1.18 (1.11: 1.26)
lag3	1.2 (1.13: 1.27)	1.05 (1: 1.1)	1.01 (0.97: 1.06)	1.09 (1.03: 1.17)	1.18 (1.11: 1.26)	1.05 (1: 1.1)	1 (0.96: 1.05)	1.07 (1: 1.14)
lag4	1.1 (1.02: 1.18)	1.03 (0.98: 1.09)	0.98 (0.93: 1.04)	1.01 (0.94: 1.09)	1.09 (1.01: 1.17)	1.04 (0.98: 1.1)	0.97 (0.92: 1.03)	0.99 (0.91: 1.08)
lag5	1.04 (0.97: 1.12)	1.02 (0.97: 1.08)	0.97 (0.91: 1.02)	0.97 (0.90: 1.05)	1.03 (0.96: 1.11)	1.03 (0.97: 1.08)	0.96 (0.91: 1.01)	0.96 (0.88: 1.04)
lag6	1.03 (0.97: 1.09)	1.02 (0.97: 1.07)	0.97 (0.93: 1.02)	0.97 (0.91: 1.04)	1.02 (0.95: 1.08)	1.02 (0.97: 1.07)	0.96 (0.92: 1.01)	0.96 (0.9: 1.03)
lag7	1.04 (0.98: 1.09)	1.02 (0.97: 1.06)	0.99 (0.95: 1.03)	1.00 (0.95: 1.06)	1.02 (0.97: 1.08)	1.01 (0.97: 1.06)	0.98 (0.94: 1.02)	0.99 (0.93: 1.06)
lag8	1.06 (1: 1.13)	1.02 (0.97: 1.07)	1.01 (0.96: 1.06)	1.04 (0.97: 1.11)	1.05 (0.98: 1.12)	1.01 (0.96: 1.06)	1.01 (0.96: 1.06)	1.03 (0.96: 1.11)
lag9	1.08 (1.01: 1.17)	1.02 (0.96: 1.07)	1.03 (0.97: 1.09)	1.08 (1.00: 1.17)	1.07 (0.99: 1.15)	1.01 (0.95: 1.07)	1.03 (0.97: 1.09)	1.07 (0.99: 1.17)
lag10	1.09 (1.01: 1.17)	1.01 (0.96: 1.07)	1.05 (0.99: 1.1)	1.10 (1.02: 1.19)	1.07 (1: 1.16)	1 (0.95: 1.06)	1.05 (0.99: 1.11)	1.1 (1.01: 1.19)
lag11	1.08 (1.01: 1.15)	1.01 (0.96: 1.05)	1.06 (1.01: 1.11)	1.11 (1.04: 1.18)	1.06 (1: 1.13)	0.99 (0.95: 1.04)	1.06 (1.01: 1.11)	1.1 (1.03: 1.18)
lag12	1.05 (0.99: 1.12)	1 (0.95: 1.04)	1.06 (1.01: 1.11)	1.10 (1.03: 1.17)	1.04 (0.98: 1.11)	0.98 (0.94: 1.03)	1.06 (1.01: 1.11)	1.09 (1.02: 1.16)
lag13	1.02 (0.93: 1.11)	0.99 (0.92: 1.05)	1.06 (0.99: 1.13)	1.08 (0.98: 1.18)	1.01 (0.92: 1.11)	0.98 (0.91: 1.04)	1.05 (0.99: 1.12)	1.07 (0.97: 1.17)
lag14	0.98 (0.85: 1.12)	0.97 (0.88: 1.08)	1.06 (0.96: 1.17)	1.05 (0.92: 1.21)	0.98 (0.85: 1.12)	0.97 (0.87: 1.07)	1.05 (0.95: 1.16)	1.04 (0.89: 1.2)

**Table 4:** Relative risk (RR) estimates and 95% confidence intervals for preterm births and Apparent temperature. All RRs are relative to median value of apparent temperature distribution (11.4 °C)

	Model 1				Model 2 (additionally adjusted for air pollution)			
	P1	P25	P75	P99	P1	P25	P75	P99
lag0	1.64 (1.47: 1.84)	1.11 (1.03: 1.21)	1.25 (1.15: 1.36)	1.61 (1.41: 1.83)	1.61 (1.45: 1.8)	1.11 (1.03: 1.21)	1.26 (1.16: 1.37)	1.55 (1.36: 1.77)
lag1	1.45 (1.35: 1.56)	1.09 (1.03: 1.15)	1.16 (1.1: 1.22)	1.4 (1.29: 1.52)	1.43 (1.33: 1.54)	1.09 (1.03: 1.15)	1.16 (1.1: 1.22)	1.36 (1.25: 1.48)
lag2	1.3 (1.24: 1.36)	1.07 (1.03: 1.11)	1.07 (1.04: 1.11)	1.23 (1.16: 1.3)	1.27 (1.21: 1.34)	1.07 (1.03: 1.11)	1.07 (1.03: 1.11)	1.2 (1.13: 1.27)
lag3	1.17 (1.12: 1.24)	1.05 (1.01: 1.09)	1.01 (0.97: 1.05)	1.1 (1.04: 1.17)	1.15 (1.1: 1.22)	1.05 (1.01: 1.09)	1 (0.96: 1.04)	1.08 (1.01: 1.15)
lag4	1.09 (1.02: 1.16)	1.03 (0.98: 1.08)	0.96 (0.92: 1.01)	1.01 (0.94: 1.09)	1.07 (1.01: 1.14)	1.03 (0.99: 1.08)	0.96 (0.91: 1)	1 (0.93: 1.08)
lag5	1.04 (0.98: 1.11)	1.02 (0.97: 1.06)	0.94 (0.9: 0.99)	0.97 (0.9: 1.04)	1.03 (0.97: 1.09)	1.02 (0.98: 1.07)	0.94 (0.89: 0.98)	0.96 (0.89: 1.04)
lag6	1.03 (0.98: 1.09)	1.01 (0.97: 1.05)	0.94 (0.91: 0.98)	0.96 (0.9: 1.02)	1.02 (0.97: 1.07)	1.01 (0.97: 1.05)	0.94 (0.9: 0.98)	0.96 (0.9: 1.02)
lag7	1.04 (1: 1.09)	1 (0.97: 1.04)	0.96 (0.93: 1)	0.98 (0.93: 1.03)	1.03 (0.98: 1.08)	1 (0.97: 1.04)	0.96 (0.93: 0.99)	0.98 (0.93: 1.04)
lag8	1.06 (1.01: 1.12)	1 (0.96: 1.04)	0.99 (0.96: 1.03)	1.01 (0.95: 1.08)	1.05 (1: 1.11)	1 (0.96: 1.04)	0.99 (0.95: 1.03)	1.01 (0.95: 1.08)
lag9	1.08 (1.02: 1.15)	1 (0.95: 1.05)	1.02 (0.98: 1.07)	1.05 (0.98: 1.13)	1.08 (1.01: 1.15)	1 (0.95: 1.04)	1.02 (0.98: 1.07)	1.05 (0.97: 1.13)
lag10	1.09 (1.02: 1.16)	1 (0.95: 1.04)	1.05 (1: 1.1)	1.08 (1: 1.16)	1.08 (1.02: 1.15)	0.99 (0.95: 1.04)	1.05 (1: 1.1)	1.07 (0.99: 1.16)
lag11	1.08 (1.02: 1.14)	1 (0.96: 1.04)	1.06 (1.03: 1.11)	1.09 (1.02: 1.16)	1.07 (1.02: 1.13)	0.99 (0.95: 1.03)	1.07 (1.02: 1.11)	1.08 (1.01: 1.15)
lag12	1.06 (1: 1.11)	1 (0.96: 1.03)	1.08 (1.04: 1.11)	1.09 (1.02: 1.15)	1.05 (1: 1.11)	0.99 (0.95: 1.03)	1.07 (1.04: 1.11)	1.07 (1: 1.14)
lag13	1.03 (0.95: 1.11)	1 (0.94: 1.05)	1.08 (1.03: 1.14)	1.08 (0.99: 1.17)	1.02 (0.94: 1.11)	0.99 (0.94: 1.05)	1.08 (1.02: 1.14)	1.05 (0.96: 1.15)
lag14	0.99 (0.88: 1.12)	1 (0.92: 1.08)	1.09 (1: 1.18)	1.07 (0.93: 1.22)	0.99 (0.88: 1.12)	0.99 (0.91: 1.08)	1.08 (0.99: 1.17)	1.03 (0.9: 1.18)

**Fig. 3:**
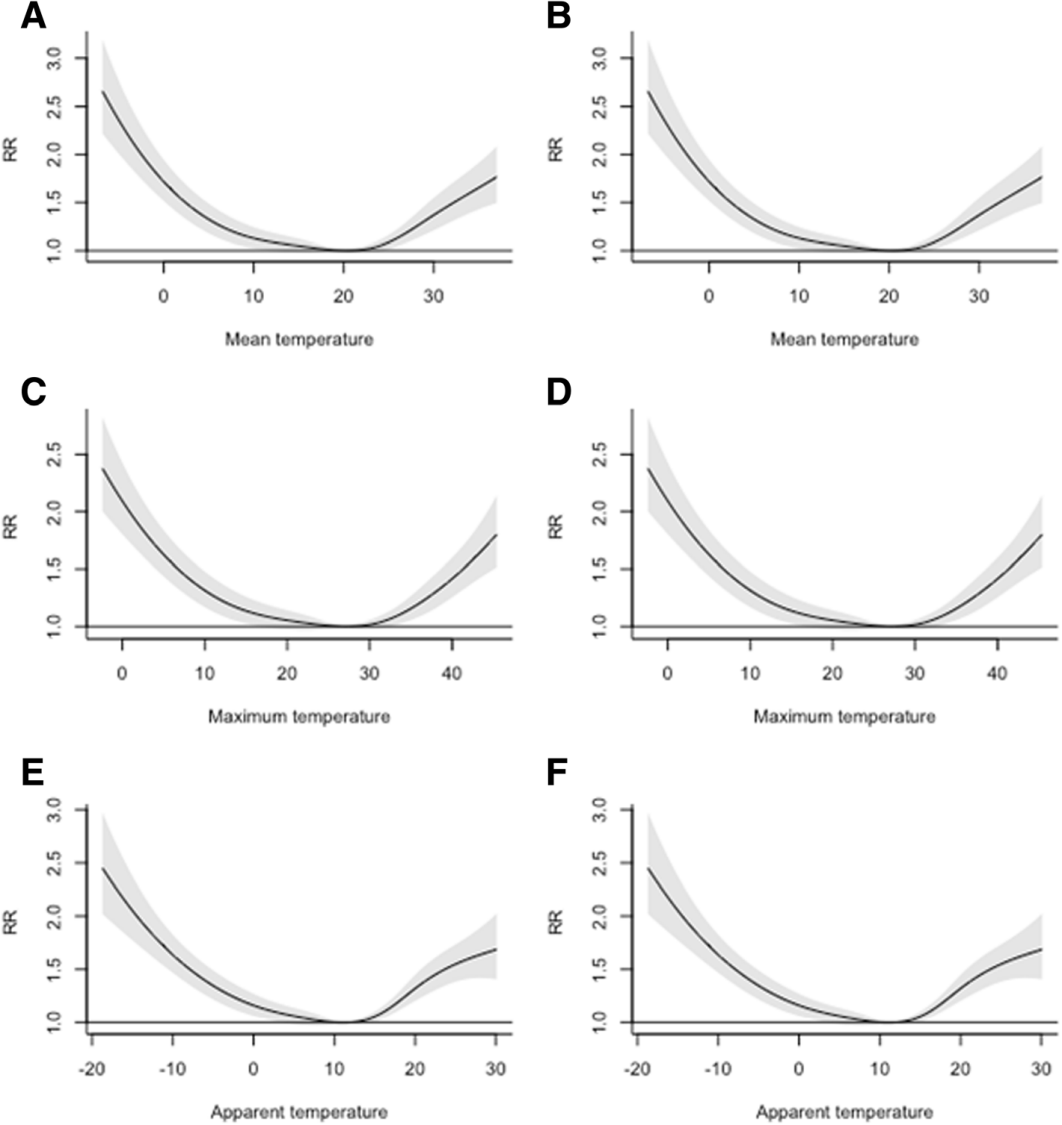
Span of significant increase in risk according to different heat stress indexes. **a**, **c**, and **e** are models without air pollution adjustment, whereas **b**, **d**, and **f** are for models with air pollution adjustment)

Exposure-response association of heat stress indexes with the relative risk of daily PTB plotted at the lag of 0 for models with and without air pollution, is shown in Fig. 3. A threshold of daily mean temperature at a hot and cold side of the observations associated with significant increase in daily PTB was different according to the heat stress index used in the model. The increase of PTB risk at the cold side was different from 16.6 °C (for maximum temperature) to 10.2 °C for apparent temperature. The increase of PTB risk started to be significant from 17.5 °C for apparent temperature till 32.4 °C for mean temperature. In a heatwave analysis, we found a significant increase in the risk of PTB in heatwave days compared to the non-heatwave days (RR 1.21; CI 1.08: 1.37). We found lower risk for days with larger temperature variation (Additional file 1: Figure S1).

## Discussion

In this study, we evaluated the possible association between exposure to hot and cold environmental temperature with a daily number of PTB in a Sabzevar city, northeastern Iran. We found an increased risk of PTB at both very hot and very cold temperatures especially at lag 0 till 8 lag days. Depending on the index used in the models, the strength of observed associations and also the threshold of temperature/index in which the risk estimates were significant, was different. Risk estimates for AT in both extremely hot and extremely cold temperatures were highest in comparison with maximum and mean temperatures. However, the observed shape of exposure-response relationship was relatively similar across different heat stress indexes.

The cyclic pattern of a daily number of PTB with the meteorological parameters of preceding and index day of delivery was reported in several previous studies [4, 11, 13]. However, nearly all of the available studies about the effect of environmental temperature and PTB are conducted in mild and temperate climates. A recent review [13] found only four studies [23–26] in Asian countries on PTB and environmental temperature. Considering the existence of the arid and Saharan area in Asian countries and on the other side the phenomenon of acclimatization of inhabitants, it is necessary to do more research on the association of temperature and preterm birth in hot and dry areas like Middle East countries [13]. To the best of our knowledge, it is the first study which is conducted in a hot and dry climate with the mean daily temperature of 26.9 °C and maximum temperature of 45.4 °C. Heat acclimation is one of the most important sources of heterogeneity in the available finding on the association between thermal stress and PTB across the globe. We had no access to possible available data about outdoor working of females in Sabzevar. However, according to the cultural context of the city, outdoor working for females is not common. Therefore, we think most of the females in the city are not acclimated especially to cold conditions. Additionally, almost all of population on the city is provided with electricity and national natural gas network connections. Therefore, using air conditioning and heating systems on summer and winter is common. Additionally, most of the women especially in small cities of Iran are housewives and therefore the chance of outdoor exposure to heat and cold stress is reduced in them. All of abovementioned assumptions lead us to consider the population under the study as unacclimated population.

We found a significant increase in the risk of PTB in elevated temperature in the population under study. The precise mechanism(s) governing PTB due to short-term exposure to hot and cold environments is poorly understood. However, stimulation of secretion of antidiuretic and oxytocin hormones due to dehydration and subsequent uterine contractility could be an explanation [23]. In contrast with our finding, a study in China found a protective effect of hot temperature on risk of PTB [24]. Several explanations could be available for this finding. Firstly, it seems that at communities with high socioeconomic status, people are using more heating, ventilating, and air conditioning (HVAC). Therefore, they can suppress the effect of outdoor temperature on their bodies. Level of education and health literacy of people are also important. Those who are more educated probably will exercise a more protective behavior and therefore lower their exposure to the heat.

We found an increase in the risk of preterm birth in both hot and cold periods. For both extremely hot and cold temperatures, the highest heat effect was observed at lag 0. It is in accordance with other studies which found the highest risk for exposure to heat stress was at lag 0 [27]. Our risk estimates in this study were relatively higher than the values reported by other studies. A study in the USA found 12 to 16% increase in the risk of PTB for 2.8 °C increase in a temperature during the week preceding delivery in the warm seasons [28]. There are several other studies which found no significant association between temperature and preterm birth [14–16]. However, it seems that the results are dependent on the geographic location of the study area. For example, a result of a study conducted in Italy [29] was different from the results obtained from the study in London [30].

We found the highest risk increase for extremely cold temperatures in all models. The available evidence about the association between cold exposure and preterm birth is relatively limited in comparison with hot temperatures [23]. Our finding is in accordance with He et al. [23] which found an increase in the risk of PTB at both cold and hot temperatures. However, some other studies reported a protective effect of elevated temperatures on PTB [24, 26]. Two other studies in Rome, Italy [31], and London, the UK [31], find no significant association between exposure to cold conditions and preterm births; conversely, a significant association for elevated temperatures was found. The winter in the abovementioned cities is mild and could be regarded as a possible explanation of the contradictory finding between our study and these studies. There are several possible hypotheses about the possible causal association between cold temperatures and preterm birth. The cold environment could affect pregnancy outcomes because of changes in pregnant women’s activity patterns during winter, increased rates of infectious diseases, or increase in the prevalence of pregnancy-induced hypertension [32]. Our finding of observed highest risk for extremely low-temperature conditions could be due to acclimatization of these people to very hot environments rather than cold conditions.

Air pollution was thought to be an intermediate in a casual pathway between temperature and preterm birth. Controlling air pollution in such studies may block part of the total effects of temperature [23]. However, we found no significant change in the risk estimates after adjusting the model for air pollution. In accordance with our study, Schifano et al. [29] also found that inclusion of air pollution in the models did not change their estimations. Mechanisms that link air pollution and environmental heat stress to preterm birth are not completely recognized. Air pollution probably pertains to the birth outcomes at both the early and final weeks of pregnancy [31]; however, the temperature might act only on the immediate final weeks and days of pregnancy [32, 33].

Use of a modified thermal environment, a different level of hydration of pregnant women, and ecologic fallacy due to loss of spatial analysis of exposure (use of only one monitoring station) can lead to error in this study. Risk of bias due to misclassification of exposure because of a thermally modified environment could be more important in high socioeconomic status populations. Confounders such as different levels of physical activity in different seasons or different levels of vaginal infection also should be considered in future studies. Effect of occupational exposure to heat stress and also housing conditions are also important factors in observed associations. However, in the area of this study, the rate of women employment is low and therefore the effect of occupational exposure to heat stress could be neglected.

Proper selection of heat stress index and the inclusion of wind speed and relative humidity in the models are an important issue in the studies on health effects of thermal stress. Environmental heat stress is a combination of the air temperature, the humidity content of the air, and the air velocity. Use of raw thermal indices such as minimum, mean, and maximum temperatures as a surrogate of thermal heat stress will omit the possible effects of relative humidity and air velocity. However, one other study found no association between PTB and change in relative humidity [26]. We also found the best model fitting index (according to CVS) at the models which used AT as a surrogate of heat stress. Our findings in higher risk estimate for apparent temperature which is composed of all of these abovementioned factors are in accordance with this property of apparent temperature.

Despite its uniqueness because of the range of temperature considered in this paper, our study suffers from several limitations. We were unable to consider the effect of a personal and indoor modifying environment and occupational heat stress in our models. Improved housing conditions and indoor occupation can alleviate the effects of outdoor temperature and bad weather. The study does not include the effect of heat exposure in the workplace and home or use of air conditioning. Like other ecological studies, we used readings from one monitoring station in the city. These data suffer from lack of spatial resolution and consideration of the effect of city heat islands. It could introduce misclassification due to a measurement error especially in the more urban area which hosts thermal islands. In this study, we did not include the age of pregnant women as a covariate in the models. It is also a limitation in this study. However, because we recruited the data from several years and with this assumption that the age of pregnant women in the city did not change in the span of the analysis, this problem can be tackled. In addition, to the best of our knowledge, none of the available studies has established causal links between environmental temperature exposure and PTB due to the limitations of their study design.

## Conclusion

In estimating the health effects of heat stress, the proper index should be selected. The effect of heat stress on PTB might be separate from air pollution. People in the arid area are not acclimatized to cold temperatures, and therefore they are more prone to the effect of low temperatures in comparison with high temperatures. Further studies should focus on cold temperatures’ effect on birth outcomes. Healthcare workers and obstetricians working in the arid area should be educated and be alert about the unwanted health effects of hot and cold environmental temperatures on the pregnant women.

## Additional file

Additional file 1: Preterm birth risk according to daily temperature variation. (PNG 20 kb)

## Abbreviations

AT: Apparent temperature
CI: Confidence interval
CVS: Cross-validation score
dfs: Degrees of freedom
dlnm: Distributed lag non-linear model
DOW: Day of week
GAM: Generalized additive model
HIS: Health information system
HVAC: Heating, ventilating, and air conditioning
ICD: International Classification of Diseases
LMP: Last menstrual period
PTB: Preterm birth
RR: Relative risk
VP: Vapor pressure
