# Supplementary figures and images for: Environmental extreme temperature and daily preterm birth in Sabzevar, Iran: a time-series analysis
Source: Environ Health Prev Med. 2019 Jan 5;24:5. doi: 10.1186/s12199-018-0760-x (PMC6320631; doi:10.1186/s12199-018-0760-x)

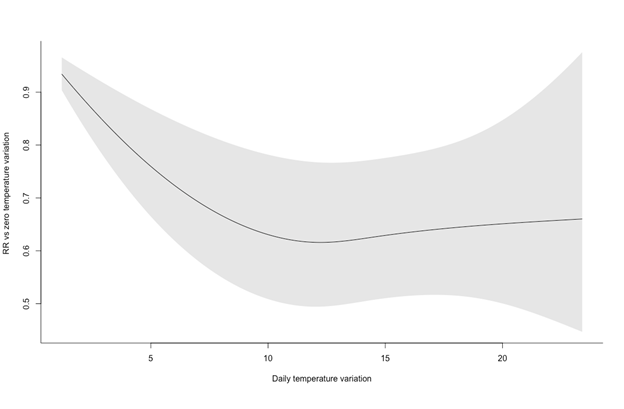

Supplement: Supplementary file 1 — Preterm birth risk according to daily temperature variation. (PNG 20 kb) [file 12199_2018_760_MOESM1_ESM.png]
